# Slower environmental cycles maintain greater life‐history variation within populations

**DOI:** 10.1111/ele.13867

**Published:** 2021-09-02

**Authors:** John S. Park, J. Timothy Wootton

**Affiliations:** ^1^ Committee on Evolutionary Biology University of Chicago Chicago Illinois USA; ^2^ Department of Ecology & Evolution University of Chicago Chicago Illinois USA

**Keywords:** agent‐based model, cyclical environments, evolutionary demography, fluctuating environments, individual heterogeneity, intraspecific variation, life histories, phenotypic variance

## Abstract

Populations in nature are comprised of individual life histories, whose variation underpins ecological and evolutionary processes. Yet the forces of environmental selection that shape intrapopulation life‐history variation are still not well‐understood, and efforts have largely focused on random (stochastic) fluctuations of the environment. However, a ubiquitous mode of environmental fluctuation in nature is cyclical, whose periodicities can change independently of stochasticity. Here, we test theoretically based hypotheses for whether shortened (‘Fast’) or lengthened (‘Slow’) environmental cycles should generate higher intrapopulation variation of life history phenotypes. We show, through a combination of agent‐based modelling and a multi‐generational laboratory selection experiment using the tidepool copepod *Tigriopus californicus*, that slower environmental cycles maintain higher levels of intrapopulation variation. Surprisingly, the effect of environmental periodicity on variation was much stronger than that of stochasticity. Thus, our results show that periodicity is an important facet of fluctuating environments for life‐history variation.

## Graphical abstract

Cyclical fluctuations of the environment are ubiquitous in nature (e.g. daily, tidal, seasonal cycles), and their role in shaping individual trait variations within populations is broadly relevant for ecological and evolutionary processes. Mathematical convenience has made stochasticity (randomness) the main tool for characterizing fluctuating environments. In this study we show, through a combination of individual‐based modeling and laboratory selection experiment using the copepod *Tigriopus californicus*, that periodicity of environmental fluctuations plays an important role in selectively shaping life history variation within populations.

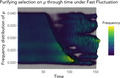

## INTRODUCTION

Individual variation in populations provides the raw material for natural selection, influences how demographic dynamics (e.g. population growth, extinction) unfold (Kendall & Fox, 2002), how multiple species interact to shape community dynamics (Bolnick et al., 2011), and how species’ responses to global change are mediated (Moran et al., 2016). Understanding the factors that generate and maintain trait variation within populations is thus a central goal of ecology and evolution. Contributions to the total phenotypic variance observed in a population are classically partitioned into genetic and environmental components, and their interactions (Bull, 1987; Falconer & Mackay, 1996; Lynch & Walsh, 1998). While the mapping of genotypic variance to phenotypic variance has been a main pillar of quantitative genetics and evolutionary studies for several decades, how environmental variance shapes phenotypic variance remains relatively less understood. Often, the environmental variance component is relegated to subsume any causally unexplained variations in the observed phenotypes, such as those that are assumed to arise from random environmental noise experienced during development and growth. When characterising environmental variance, stochasticity (random fluctuation) is typically the tool that is used. Theoretical effort has focused on exploring the different outcomes of stable versus stochastic environment assumptions for ecological and evolutionary dynamics (Coulson & Tuljapurkar, 2008; Engen et al., 2020; Koons et al., 2016; Lande et al., 2017; Metcalf & Koons, 2007; Sæther & Engen, 2015; Tuljapurkar et al., 2009; Vindenes et al., 2008; Vindenes & Langangen, 2015).

Though stochastic variables can approximate real temporal variability of environments and lend useful ecological and evolutionary insights, randomness overlooks a pervasive mode of environmental fluctuations in nature: periodic oscillations. Many environmental cycles in nature are periodically forced by a fundamental driver, such as the revolution of the Earth around the Sun that causes seasonality, or the lunar cycle that controls the tides. Stochastic models can be used to characterise environments with frequency spectra that may be indistinguishable from fundamentally cyclical but noisy environments, for example by using a random variable for event timing drawn from a Gaussian distribution with a mean periodicity and a standard deviation that scales the amount of temporal stochasticity (Lytle, 2001). Autocorrelative functions can be added to stochastic models (Metcalf & Koons, 2007; Vasseur & Yodzis, 2004; Wieczynski et al., 2018) to approximate noisy environments with some time‐lagged memory. However, the distinct influences of periodicity and noise—two important axes of environmental fluctuations—on ecological and evolutionary processes remain an important target of study. One reason is that periodicity and noise can vary orthogonally. For example, the period length of warm (‘growing’) seasons optimal for biological activity varies clinally across latitudes (shorter towards higher latitudes) independently from change in seasonal stochasticity driven by climate change (Easterling et al., 2000; Xu et al., 2013; Zhu et al., 2012). Even non‐externally forced regimes that have emergent cyclical behaviour due to internal system dynamics, such as fire, show orthogonal variations of average frequency and stochasticity (Marlon et al., 2009; Moritz et al., 2012; Westerling et al., 2006).

Models of periodic environments have shown that periodicity has significant impacts on population dynamics (Caswell & Trevisan, 1994; Tuljapurkar, 1985) and life history evolution (Park, 2019). Yet, these are concerned with population‐level properties such as optimal or mean traits. For studying the environment's role in shaping variability among individuals of a population, stochasticity remains the main conceptual framework (Bull, 1987; Canino‐Koning et al., 2019; van Daalen & Caswell, 2020). Here, we investigate how variance in life history traits within populations is influenced by environment periodicity and stochasticity separately. Specifically, we consider the case where environmental fluctuations are on similar timescales as generation time, population size is free to change and generations overlap.

**FIGURE 1:**
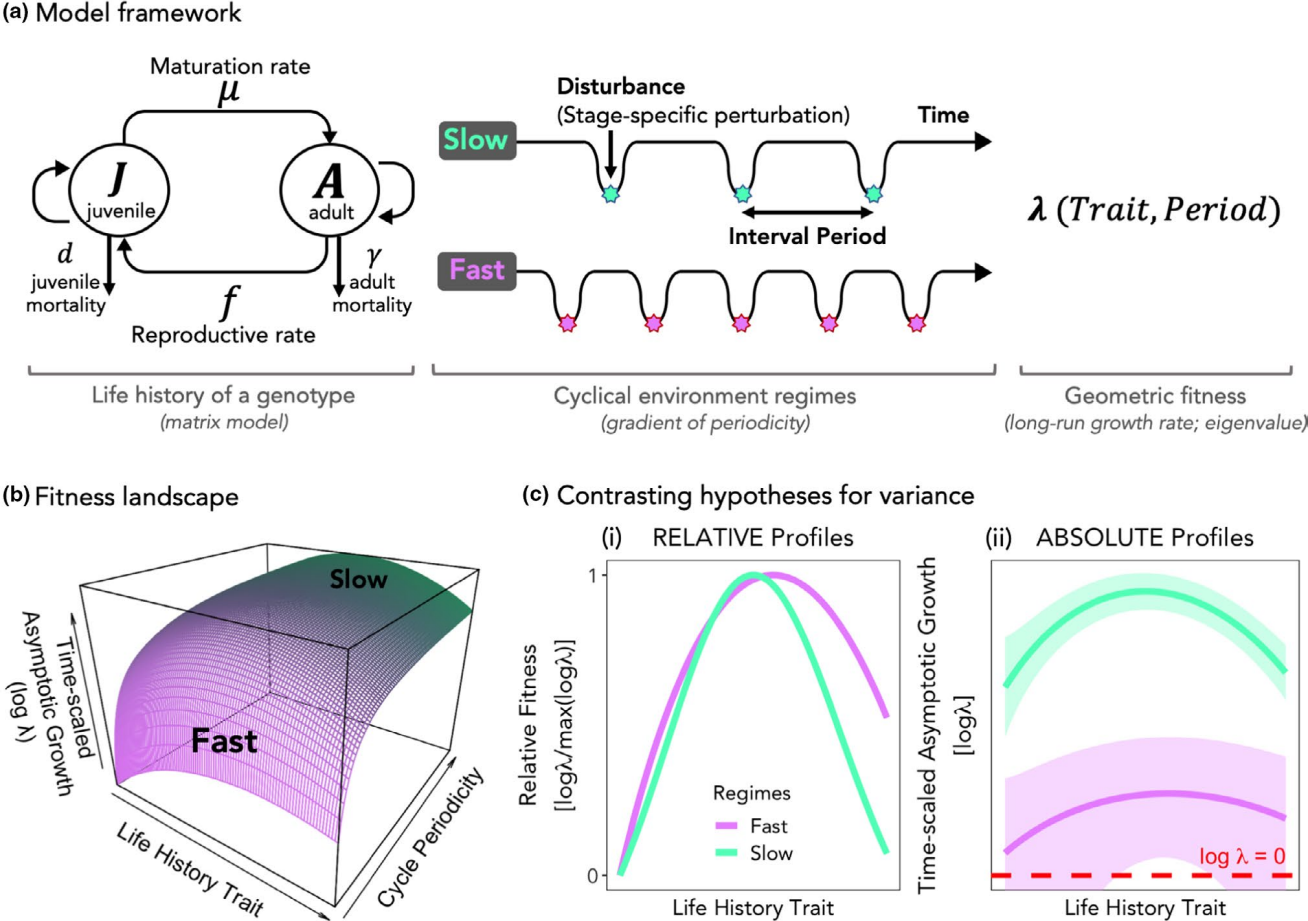
Optimality model of life histories in cyclical environments, and contrasting hypotheses for variance. (a) The deterministic theoretical model developed in Park (2019) takes the life‐history strategy of a genotype—which consists of life‐history traits and trade‐offs among them—and calculates the asymptotic growth rate (dominant eigenvalue λ of the matrix model) as a continuous function of traits and environmental periodicity (see Data S1 for abridged derivation). (b) The resulting landscape gives optimal life histories (peaks on trait axis) across the axis of environmental cycle periodicity. Note that asymptotic growth rate on the *z*‐axis is scaled by matrix projection interval which varies along the cycle periodicity axis. (c) Here we take two near‐extreme cases of periodicity, namely “Fast” (low period) and “Slow” (high period), and investigate contrasting hypotheses for observed trait distributions. (i) Relative (to the peak in each regime) profiles show greater width under the Fast regime, suggesting weaker selection leading to larger variance than under the Slow regime. However, (ii) the absolute profile of asymptotic growth under the Slow regime is overall higher than that under the Fast regime, simply due to less frequent disturbances in the Slow regime. The hypothetical bands represent uncertainties in realised growth due to drift, which inversely scales with population size. In this alternative perspective, lower variance is expected in the Fast environment because, given drift, many genotypes are likely to cross the line of log λ = 0 (zero growth) which advances extinction

Two hypotheses for variance are given by a recent theoretical fitness landscape model of life histories in cyclically disturbed environments (Park, 2019). In this deterministic framework (Figure 1a), the asymptotic growth rate of a genotype is given as a function of its life‐history traits, trade‐offs among them and the periodicity of the environment, calculated as the dominant eigenvalue (λ) of the underlying vital rates in a matrix model (see Data S1 for derivation). As the main output, a fitness landscape is given by scanning across life histories and environment periodicities (Figure 1b). Here, we ask how the topography of the landscape relates to the observed variance of traits across environmental periodicity. We take two representatives from the periodicity spectrum, henceforth “Fast” (low period) and “Slow” (high period) environments. On one hand, relative log λ (scaled to maximum log λ in each regime) shows a sharper profile in the Slow regime (Figure 1c(i)), which might indicate stronger stabilising selection and thus lower variance compared to the Fast regime. On the other hand, when seen in absolute terms (time‐scaled by matrix projection interval for a balanced comparison), the entire profile in the Slow regime consists of higher magnitudes of asymptotic growth rate compared to the Fast (Figure 1c(ii)), which indicates high persistence of all genotypes. In other words, even suboptimal genotypes in slow environments can proliferate and remain in the population in high numbers due to less frequent disturbances. Contrastingly, in Fast environments, all genotypes are much closer to log λ < 0, which advances extinction (Figure 1c(ii)). This discrepancy in profile heights presents an interplay between natural selection and drift: natural selection governs the relative survivability between genotypes, but drift, whose effect inversely scales with population size, influences how many genotypes are at risk of extinction (Figure 1c(ii)) especially at the tails of distributions, which would reduce variance in the population. While these profiles constructed with deterministic log λ’s do not equate to growth in stochastic environments, the general height difference between profiles due to more or less frequent disturbances should hold in stochastic analogues, and thus motivates our study of deterministic and stochastic cases of Fast and Slow environments.

Here, we test these two hypotheses—higher variance expected in Fast environments due to a weaker selection profile *versus* higher variance in Slow environments due to broad proliferation of suboptimal genotypes—with an agent‐based (also known as individual‐based) model of life history evolution in cyclically and stochastically disturbed environments. We further corroborate our simulations with a life history selection experiment in the lab. We used the copepod *Tigriopus californicus* found in periodically disturbed tidepools of the Pacific Northwest, USA to parameterise our simulations and conduct the experiment. We explicitly differentiate environmental periodicity and stochasticity, and show that these two subcomponents of environmental fluctuations have distinct contributions to intraspecific life‐history variation, and that periodicity plays a surprisingly large role.

## METHODS

### Demography and life history of *Tigriopus californicus*

The copepod *T. californicus* forms dense populations typically reaching thousands of individuals in <1 L small rock pools above the marine intertidal zone along the Pacific coastline of North America (Dethier, 1980; Powlik, 1999). Individuals develop through distinct stages from juveniles to reproducing adults. After mating, females produce a series of clutches every few days until death; ‘egg‐to‐egg’ generation time ranges between 20 and 30 days (Burton et al., 1979). Pools containing populations experience wave disturbance from the tide cycle at measurably consistent periodicities depending on the height of the pool relative to the tide line; this periodicity across populations ranges from 2–3 days to two weeks (Park, 2019). Whenever populations are disturbed periodically by waves at high tide, *T. californicus* cling to the benthos of their pools to avoid being washed out; yet many are dislodged to the lower intertidal zones where predators such as sculpin feed on them quickly (Dethier, 1980; Dybdahl, 1995). These disturbances decrease population size periodically, but more importantly they incur stage‐specific mortality by killing more juveniles than adults (Park, 2019). We used extreme representatives of periods measured in nature to parameterise the Fast (period = 3 days) and Slow (Period = 14 days) cycle regimes in both the simulations and experiment.

### Agent‐based model (ABM) of life history evolution in fluctuating environments

We simulated selection dynamics in populations containing varying life history traits. We subjected these in silico populations, parameterised for the *T. californicus* system, to deterministically cyclical, and stochastic fluctuation regimes and tracked *i*‐state configurations (developmental states of individuals) through time. Then we analysed the distributions of life history traits of the surviving genotypes. See Data S2 for schematic of the IBM.

#### Birth, growth and death of individuals

We initiated each population with a set of *n* genotypes $i=\left\{1\ldots n\right\}$, each defined by two life history phenotypes: maturation rate $\left\{\mu_{1},\ldots ,\mu_{n}\right\}$ and fecundity $\left\{f_{1},\ldots ,f_{n}\right\}$. Each individual progressively grows through continuous states $s_{i}=\left[0,3.0\right]$, transitioning through life‐history stages where 0 = new‐born, [0, 1.0) = reproductively immature juvenile, [1.0, 3.0) = reproductively mature adult and 3.0 = end of life. At the beginning of each simulation, the population begins at *n* = 5000, and consists of a 25:1 ratio of juveniles:adults, which was the approximate steady‐state stage‐structure from exploratory simulations, but initial states within each stage are randomly drawn from a uniform distribution. Then each individual grows ($s_{i}$ updated) at increments of $\mu_{i}$ per time‐step. Therefore, genotypes reach reproductive maturity in different amounts of time, and spend different amounts of time as reproductive adults. As soon as juveniles reach $s_{i}=1.0$, they begin reproducing continuously following a Poisson process with rate $f_{i}$. This continuous manner of reproduction is a good approximation of the reproductive pattern of *T. californicus*. Maturation rate $\mu_{i}$ and fecundity $f_{i}$ are linearly negatively correlated, denoting a trade‐off in *T. californicus* (Dybdahl, 1995; Hong & Shurin, 2015) and parameterised from (Park, 2019) such that $f_{i}=1/\left(4*\mu_{i}\right)$. The set of initial μ’s spanned [0.02,0.04] and f’s spanned [6.25,12.50], which represent the ranges of previously measured values of these traits (Park, 2019). When a mature individual reproduces, it appends a number of offspring to the population vector, where number ~Pois($f_{i}$). All offspring begin life at $s_{i}=0.$ Note that the adults modeled are more precisely female adults, which is the convention in stage‐structured demographic models of two‐sex populations (Caswell, 2000). Nevertheless, recombination from mating, along with mutations and plasticity, can cause imperfect inheritance of parental traits. Thus, $\mu_{i}$ and $f_{i}$ of the newborns are copies of those of their parent plus a small error drawn from a normal distribution with mean 0 and variance 0.01*$\bar{\mu }$ for maturation and $0.01*\bar{f}$ for fecundity, where $\bar{\mu }$ and $\bar{f}$ are global means from the initial sets. While parent–offspring correlations for these specific traits in *T. californicus* have not been measured to our knowledge, we chose a reasonable error rate given heritabilities of other *T. californicus* life‐history traits (Edmands & Harrison, 2003; Kelly et al., 2012; Voordouw & Anholt, 2002).

There are four ways in which mortality occurs. First, juvenile maturation rate $\mu_{i}$ and adult fecundity $f_{i}$ trade‐off with juvenile and adult survival probabilities (i.e. positively covary with mortality), respectively. These trade‐offs are not only central pillars of general life history theory (Cohen et al., 2017; Stearns, 1989), but have been previously measured in *T. californicus*. Mortality of juveniles $\left(s_{i}<1.0\right)$ is a Poisson process with rate $5\times \mu_{i}$(maturation rate), and mortality of adults $\left(s_{i}>1.0\right)$ is a Poisson process with rate $0.001\times f_{i}$(fecundity), parameterised following (Park, 2019). Second, density‐dependent mortality is applied to all individuals regardless of state. Density‐dependent mortality per time‐step is a non‐linear function of density $N_{t}$, where $N_{t}\times 0.001\times \left(1+\frac{N_{t}}{250,000}\right)$ random individuals are excised from the population per time‐step. Minimum background mortality rate of 0.001 in optimal conditions and scaling factor of 250,000 assuming a typical 10 L *Tigriopus* pool were estimated using previously reported natural densities (Dybdahl, 1995; Hong & Shurin, 2015; Powlik, 1999). Third, when an individual reaches state $s_{i}=3.0$, it reaches end of life and dies. Finally, environmental disturbance, either periodic or stochastic, removes a random 30% of juveniles $\left(s_{i}<1.0\right)$ from the population.

#### Environmental fluctuation regimes

We evolved these *in silico* populations under four different environmental fluctuation regimes: deterministic Fast cycles (periodicity = 3 time‐steps), deterministic Slow cycles (periodicity = 14 time‐steps), and stochastic analogues of each wherein disturbance timings were drawn randomly from a uniform distribution, $\text{timing}∼U\left(0,T\right)$ where T=length of simulations, but such that the total number of disturbance events was equivalent to the deterministic analogues so that total mortality would be comparable. All rates were parameterised on the daily scale to match the laboratory experiment described below.

#### Phenotypic variance

We simulated 100 realisations of evolving populations within each of the four fluctuation regimes. For each realisation we tracked *i*‐state configurations of individuals in the population, and the distributions of maturation rates $\left\{\mu_{1},\ldots ,\mu_{n}\right\}$ and fecundities $\left\{f_{1},\ldots ,f_{n}\right\}$ in the population. We ran each simulation for 150 time‐steps, which is equivalent to 150 days or 5–6 generations of *T. californicus*, equal to the length of the lab experiment.

### Multi‐generational life‐history selection experiment

Prior to the experiment, we initiated a laboratory stock population of *T. californicus* collected from pool populations in northwest Washington State, USA, maintained in a 4‐L tank under a 12‐h photoperiod cycle at 20°C in a Percival growth chamber for 4 months (3–4 generations). We used a medium of 35‰ artificial seawater solution (Instant Ocean) in DI water and 0.4 g/L concentration of *Spirulina* powder for food. Food was added twice weekly ad libitum during pre‐experiment rearing. See Data S3 for a schematic of the experiment.

We initiated replicate experimental populations with 100 mating pairs and 100 juveniles each, randomly selected from the stock population. We maintained each population in 500 ml of 35‰ artificial seawater and 0.4 g/L *Spirulina*. We assigned eight replicate populations randomly to each of three treatments: deterministic Fast, deterministic Slow, or stochastic Slow. Fast replicates were disturbed every 3 days, Slow replicates every 14 days, and stochastic Slow replicates on random days dispersed by intervals drawn from a uniform distribution, but such that the total number of disturbance events during the span of the experiment was equal to that of the deterministic Slow treatment. Due to logistical constraints, we did not include a stochastic analogue of the Fast treatment. We administered disturbance in the form of juvenile‐specific mortality, emulating what occurs in nature. To do so, we designed a two‐layered cylindrical container with 200 μm mesh at the bottom of the inner container, whose gaps would let only juveniles to pass. Thus, for disturbance, we (1) extracted the meshed inner container, leaving only the juveniles in the medium, (2) thoroughly swirled and discarded 150 ml of the well‐mixed medium with juveniles to administer 30% juvenile mortality, (3) reinserted the meshed container thereby rejoining the adults with juveniles, and finally (4) refilled the container to 500mL to replenish medium and food. An equivalent amount of food was added to the deterministic and stochastic Slow replicates at the same 3‐day interval to match the replenishment rate of Fast replicates. All populations were kept in a growth chamber at 20°C, and rotated randomly within the chamber weekly.

Following 5 months of disturbance treatments, we selected 30 random gravid females from each of the 24 populations (3 treatments × 8 replicates) by visually checking for adult females with egg sacs. These gravid females were used to measure fecundity (f), and their offspring were used to measure maturation rate (μ). Each gravid female was kept in a separate 3.4 ml well containing the same medium and 0.4 g/L *Spirulina*, refreshed regularly with an eyedropper.

#### Fecundity (f) measurements

For each female the production of clutches of juveniles was monitored every 12 h. Once the first clutch was observed, all juveniles were cleared from the well immediately using a pipette, leaving the female isolated in the well again with fresh medium and food. The time gaps between clutches were thus recorded in increments of 12 h until each reproducing female had deposited up to three successive clutches. Then the mean ‘clutch interval’ per female was calculated. The size of each clutch was not measured due to logistical constraints, but a global average of 47.32 was assumed for all females which was measured in a previous study (Park, 2019). Fecundity was thus calculated as $\bar{clutch\;size}/\bar{clutch\;interval}$ per female.

#### Maturation rate (μ) measurements

We collected juveniles from the second clutch of each female with a pipette immediately after hatching. From this clutch 20 juveniles were randomly selected and put into a single 6.9 ml well containing medium and 0.4 g/L *Spirulina*. Food was replenished regularly and the siblings were allowed to mature and sib‐mate; this ensured that all individuals in each mating group started life at the same time. We monitored mating groups from each female parent and recorded when the first egg sac appeared on a female as the earliest and surest sign of sexual maturity. Immediately after a gravid female appeared we removed her from the well to avoid counting subsequent clutches of the same female. Then we monitored each well in 12‐h increments until two gravid females appeared in total per well, and calculated the mean age at sexual maturity of the offspring produced per original parental female.

### Statistical analyses

At the endpoints of the simulations, we analysed if intrapopulation variances of phenotypes ($\sigma_{\mu }^{2}$ and $\sigma_{f}^{2}$ within each realisation) varied between environmental fluctuation regimes with a series of Welch's two‐sample *t* tests after evaluating normality using log‐transformed variances assuming unequal size and variance.

For the experiment, we similarly calculated phenotypic variance within each population at the end of the selection period. To accommodate the non‐normal distributions and unequal numbers of phenotype measurements in replicate populations, we conducted a Monte Carlo permutation test to analyse differences in intrapopulation variances between any pair of treatments A and B. First, we randomly sampled groups of 30 individual‐level phenotypic measurements from the global dataset containing the measurements across all treatments and all replicates. We selected *N* such samples, where *N* = *a* + *b*, and *a* = number of replicates in treatment A, and *b* = number of replicates in treatment B. We calculated the variances of these *N* samples, which comprised the universal probability density function (PDF) central to such permutation tests. From the universal PDF we then randomly sampled, with replacement, the same number of variance estimates as there were replicates per treatment, and calculated *mean*(*b* variance samples) – *mean*(*a* variance samples). We permuted this calculation 50,000 times, which formed the null distribution of variance estimate differences between treatments. To test the hypothesis that the observed intrapopulation variances differed between any two experimental treatments, we computed the empirical observed difference = mean(variance measurements of replicates in Treatment B) – *mean*(variance measurements of replicates in Treatment A), and computed *p*‐value = proportion of the permutation distribution ≥ observed difference (Data S4). We conducted this permutation hypothesis test to compare Slow versus Fast, and deterministic versus stochastic Slow intrapopulation variance differences, for both μ and f.

## RESULTS

### Shifting distributions in simulated populations during transience

**FIGURE 2:**
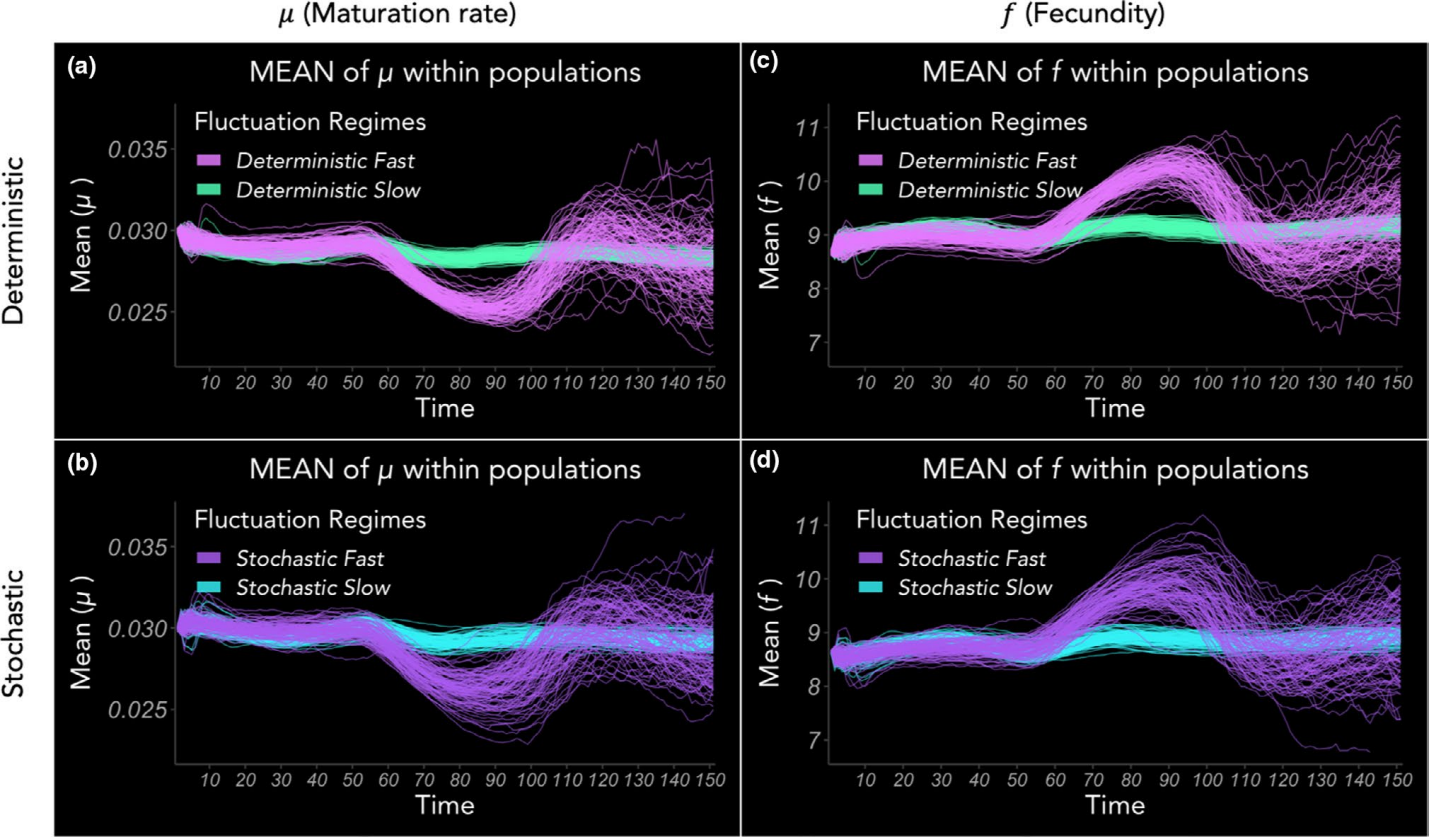
Means of life‐history traits evolving in simulated populations. (a, b) show evolving mean of μ (maturation rate) in deterministic and stochastic regimes, respectively; (c, d) show evolving mean of *f* (fecundity) in deterministic and stochastic regimes, respectively. Each scenario is repeated for 100 realisations

**FIGURE 3:**
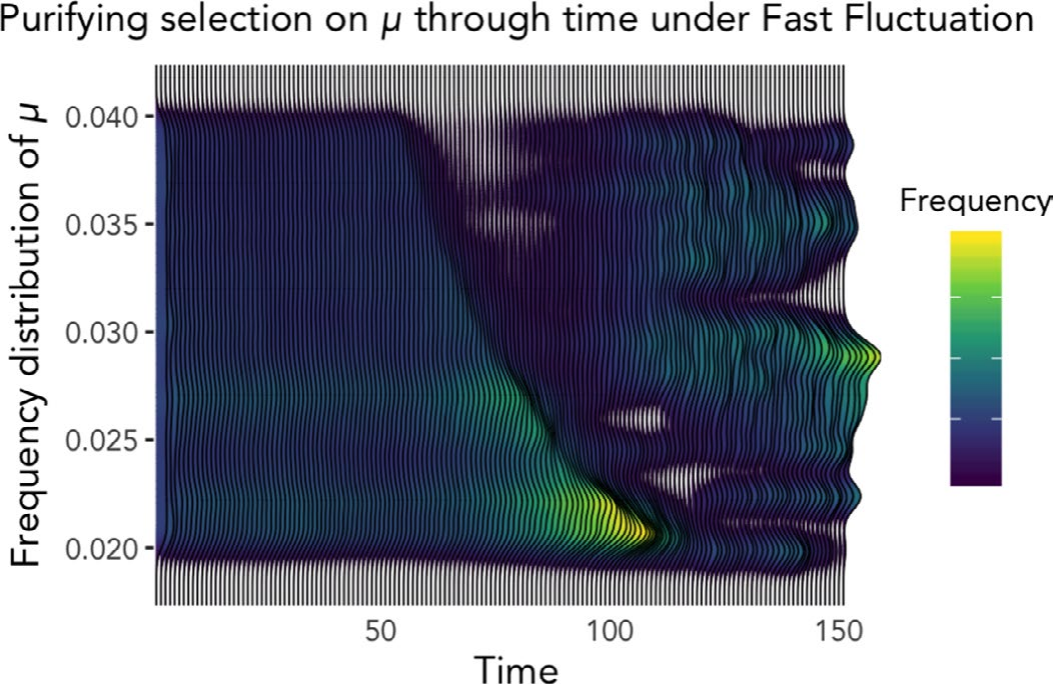
Purifying selection in action. Morphing distribution of μ (maturation rate) in one example realisation of a population under Fast environmental fluctuation shows early selection towards lower μ; this reflects short‐term benefit for fast reproducers (high *f*) that are simply adding more offspring that resemble them quickly. Thereafter, costs associated with high reproduction allow alternate phenotypes to resurge in frequency (~*t* = 75), which momentarily raises population mean as well as variance. Soon, the distribution narrows towards an optimum and variance declines (yellow peak at *t* = 150)

In all evolutionary simulation realisations, the within‐population means of maturation rate (Figure 2a,b) and fecundity (Figure 2c,d) both showed concordant patterns during the first few generations. Particularly, under all environmental fluctuation regimes, there was an initial shift towards higher mean *f* due to a short‐term proliferation of fast reproducers and, correspondingly, lower mean μ, reflecting the trade‐off between the two (Figure 3). The relative stall prior to this shift occurs because the multiplicative process of faster reproducers proliferating is less noticeable over just one generation, and simultaneously some decline in population size is needed to show pronounced changes in distributions. This shift, which was more pronounced in Fast environments due to higher juvenile mortality from more frequent disturbances, was counteracted in 1–2 generations by costs associated with high reproduction, causing a resurgence in frequency of faster‐maturing (higher μ) genotypes. This compensatory trend reflects an inter‐generational consequence of a short‐term benefit of life histories. Then, approaching the end of the simulations, approximately after 5–6 generations, the distributions began to narrow (Figure 3). Deterministically periodic regimes and their stochastic analogues (same number of disturbance events but randomly dispersed) produced qualitatively very similar trends over time (Figure 2a vs. Figure 2b, and Figure 2c vs. Figure 2d), showing that mean periodicity had a stronger influence on life‐history dynamics than temporal stochasticity of the environment.

### Higher variance in Slow simulated environments

**FIGURE 4:**
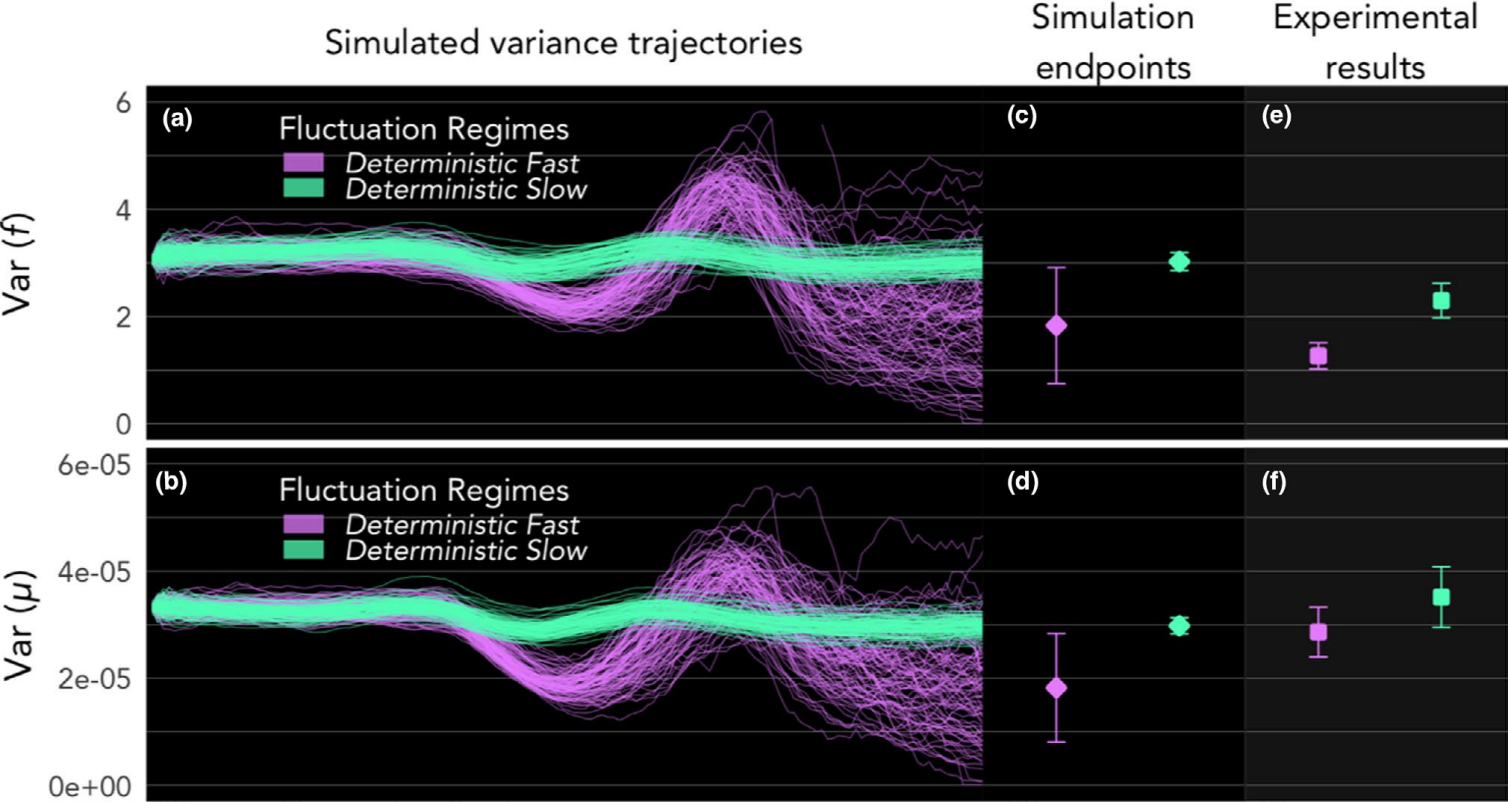
Higher phenotypic variance in Slow environments in simulation and in experiment. (a, b) Simulated trajectories of intrapopulation variance over 150 time‐steps (5–6 generations) exhibit common qualitative patterns of (1) dropping early on because fast reproducers proliferate quickly (by definition) and distributions become concentrated at high *f* and low μ values; (2) briefly spiking as alternate phenotypes rise in frequency due to life‐history costs associated with high *f* and low μ; and (3) beginning to gradually decline. (c, d) Intrapopulation variances at simulation endpoints are higher in Slow environments than Fast for both traits. Diamond points show means of variances in realisations (i.e. means of intrapopulation variance), and error bars the standard deviation among the realisation variances. (e, f) Measurements of intrapopulation phenotypic variances after the 150‐day experiment (5–6 generations) also showed higher variance in Slow treatments for both traits. Square points show means of intrapopulation variances among replicate populations, and error bars the standard error of the variance measurements

Life history variances were distinctively higher in Slow compared to Fast regimes at the end of the simulation period, for both traits (Figure 4; Figure S6). Variance trajectories over the first few generations were driven by the same processes that drove concordant shifts in trait means, namely the early proliferation of high *f* phenotypes (producing the initial decrease in variance), followed by compensatory resurgence of alternate phenotype frequencies due to costs on extreme phenotypes (producing the momentary spike in variance), before the gradual decline in variance tracking purifying selection (Figure 3). This decline was much faster under Fast fluctuations than under Slow (Figure 4a,b). Comparing the endpoints of simulations (Figure 4c,d; Figure S6.2; Table S6.1), intrapopulation variances of both traits were dramatically higher in Slow environments, for both deterministic and stochastic cases. Contrastingly, variance differences between deterministic and stochastic cases for both Fast and Slow regimes were not as strong or consistent. Maturation rate showed statistically stronger signatures of variance difference between deterministic and stochastic cases than fecundity. Overall, the strongest and most consistent driver of intrapopulation trait variance was the Fast‐Slow distinction of the environment.

### Experimental corroboration of higher variance in Slow environments

At the end of the *T. californicus* selection experiment, intrapopulation means of maturation rate and fecundity did not show consistent signatures of statistical differences (Data S5), similar to simulation results. Intrapopulation variances, however, as in the simulation, were distinctively higher in the Slow cycle treatments than in the Fast for both traits (Figure 4e,f; Table 1 ). Variances in the stochastic Slow treatments were not different from those in the deterministic Slow treatments, for both traits. Thus, there was strong evidence that periodicity drove differences in intrapopulation variance much more strongly than the deterministic‐stochastic environment distinction, for both traits.

**TABLE 1:** Comparing intrapopulation phenotypic variances among experimental populations

Trait	Experimental treatment variance comparison	*p*‐value
***f*** (fecundity)	**Deterministic Fast versus Deterministic Slow**	.**005**
	Deterministic Slow versus Stochastic Slow	.244
μ (maturation rate)	**Deterministic Fast versus Deterministic Slow**	.**012**
	Deterministic Slow versus Stochastic Slow	.418

Note

Hypothesis testing with Monte Carlo permutation showed that intrapopulation variances of both traits were significantly different between Fast and Slow treatments across replicate populations (*p*‐values under significance level α=0.05 in bold). However, variances under Slow and Stochastic Slow treatments were not significantly different.

### Speed of purifying selection and sustained variance in simulations

**FIGURE 5:**
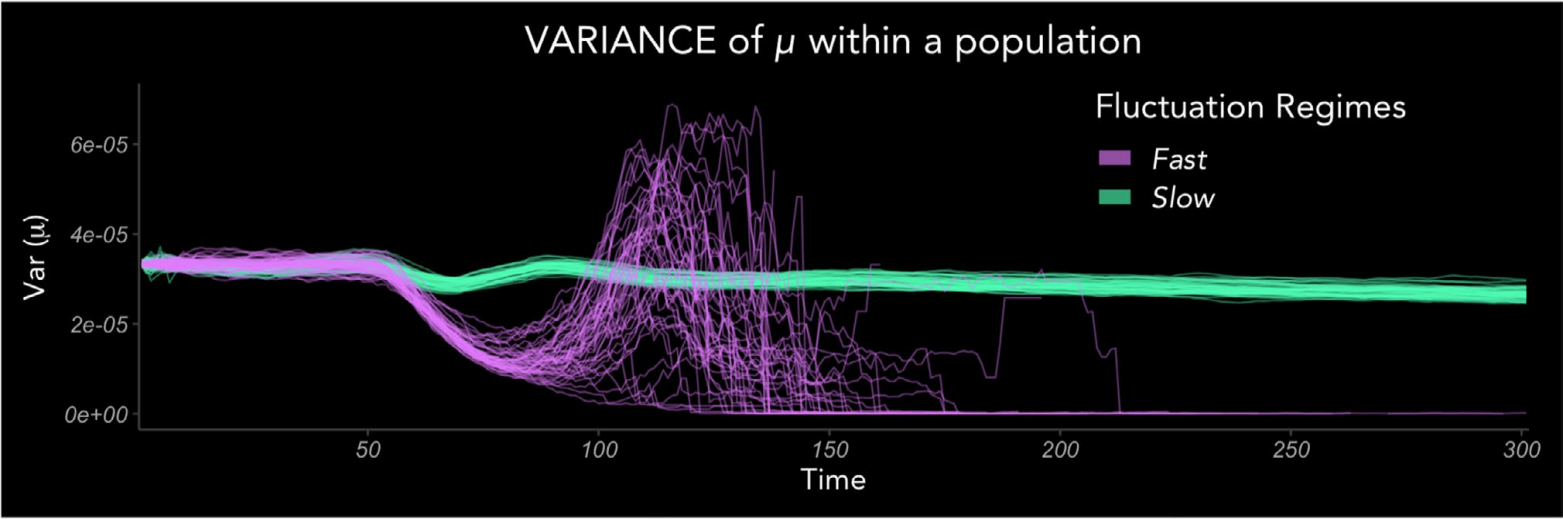
Sustained high variance in Slow environments. A longer simulation (*t* = 300; 10–12 generations) of realisations for each regime, shows that phenotypic variances of populations in Slow environments steadily decrease as well, but much more gradually than those in Fast environments. Temporary spikes of variance in the Fast regime realisations are explained by compensatory resurgence of fast growers (high μ) after short‐term dominance of fast reproducers. By ~*t* = 150, most simulated populations in the Fast regime have markedly low variance as a result of emergent purifying selection

We found that the decline in variance seen by the end of the simulation period continued when the simulation ran for longer (300 time‐steps; Figure 5), demonstrating emergent purifying selection. We found that the variance decline becomes much steadier after about 5–6 generations, as the short‐term benefits of extreme phenotypes and their time‐lagged costs eventually stabilise and the populations narrow towards optima. This decline in variance in Fast regimes was already more pronounced in the short‐term transient phase (Figure 4), and it continued to drop more precipitously than in Slow regimes in the longer simulation (Figure 5). Phenotypic variance in Slow environments declined very slowly because, despite the hypothesised stronger purifying selection (Figure 1c(i)), suboptimal phenotypes had higher λ than they would have in Fast environments (Figure 1c(ii)) and were thus sustained in the population.

## DISCUSSION

Despite a growing scope for the *consequences* of individual phenotypic heterogeneity in ecology, there is still relatively little known about the environmental *causes* that shape heterogeneity in dynamic populations (Hamel et al., 2018; Moran et al., 2016; Steiner et al., 2010). Life‐history fates are dependent on the very nature of the time sequence of the environment experienced by individuals, often periodically driven by geophysical forces, beyond broad characterisations of temporal variance captured by environmental stochasticity. Thus, life‐history variations in cyclical environments, particularly in populations with overlapping generations where individuals born at different times experience very different time sequences of the environment through their lifetimes, present a broadly relevant puzzle that highlights the interplay between natural selection and drift (Snyder et al., 2021; Tuljapurkar et al., 2020, 2021). Here we showed, through simulation and experiment grounded in evolutionary demographic fitness landscape predictions, that the periodicity of environmental fluctuations influences the level of life‐history variance in populations. Despite minor differences in effect size and spread between simulation and experiment (Figure 4)—likely due to laboratory handling or founder effects in experimental populations, among other unmeasured factors—both lines of investigation showed clear evidence of increased life‐history variation under slower environmental fluctuations.

Oscillatory behaviour of the environment, such as seasons or tides, is common in nature. We showed that the Fast‐Slow axis of environmental oscillations created dramatic differences in the level of life‐history variance in populations. Phenotypic variance is a feature of transient phases of population dynamics, under the simple assumption that every population will converge to some theoretical optimum through perfect purifying selection in the limit. Equilibrium predictions may never be reached in real populations due to ever‐present environmental and demographic stochasticity in nature, and mutations; transient dynamics are thus important for understanding and predicting the trajectory of populations (Hastings, 2004). We showed that the trajectory of phenotypic variance through transience differs radically depending on whether that population exists in a Fast (quick decline of variance) or Slow (gradual decline of variance) fluctuating environment.

We asked whether phenotypic variance would be more influenced by selection strength inferred from relative growth rates between genotypes (Figure 1c(i)), or magnitude of proliferation inferred from absolute growth rates (Figure 1c(ii)): weak selection might sustain variance, but all genotypes having high growth rates, despite stronger selection, would reduce stochastic extinctions and could sustain variance too. Within the parameter space of our investigations, the latter, conferred by longer periodicity of the environmental disturbance cycle, dramatically increased phenotypic variance despite density‐dependent mortality. Importantly, higher population size did not necessarily result in higher variance (Figure 5; Figure S7) because over a longer span of time, the optimal phenotypes eventually began to dominate via purifying selection and variance decreased despite growing population size. This suggests that the two drivers—selection strength and high overall growth rate—are indeed both at work, but the latter might be more influential for observed phenotypic variance than the former in transience. Smaller population sizes increase the effect of drift, however, which can make long‐term evolutionary outcomes more unpredictable. Indeed, we found that the spread of simulation realisations was much larger in Fast than Slow environments (Figures 2 and 4). A formal investigation of the interaction between density‐dependence and fluctuating forces of selection on life‐history traits—and how that interaction is modulated by periodicity of fluctuation—is a much‐needed direction of study.

Our work provides a testable pattern to motivate investigation of generality in other systems, for instance across species of diverse lifespans and paces of life (Salguero‐Gómez et al., 2016), and identifies key interacting mechanisms that can control patterns of individual variation in nature. Future explorations of environmental fluctuation periodicity in the context of other study species could use our model framework to evaluate how the ratio between the environmental fluctuation period and generation time of the species (e.g. period ≫ generation time, or vice versa) relates to the influence of periodicity in shaping phenotypic variance (Stearns, 1976), or demographic buffering against fluctuations (Pfister, 1998). Our framework is well‐suited to explore the role of amplitude, another important parameter of environmental fluctuations. Amplitude can be approximated by the magnitude of mortality functions in our model to systematically analyse its effect on selection strength, and consequently phenotypic variance. Amplitude would be especially important in stochastic environments where extreme events significantly reduce variation, and alter the course of subsequent selection dynamics.

Beyond expanding theoretical understanding, climate change demonstrates a clear need to study the eco‐evolutionary consequences of changing environmental cycles. While research has largely focused on the overall warming aspects of climate change, the length of the thermal growing season is consequently increasing in many ecosystems (Linderholm, 2006; Liu et al., 2018; Parmesan, 2006). This represents an expansion of annual intervals when most biological activity is optimal, or possible. Winters are conceptually analogous to periodic ‘disturbances’ and the length of intervening thermal growing seasons the ‘periodicity’ of that cycle, particularly in high latitude ecosystems. Conversely, longer summers represent a contraction of the biological window for winter‐adapted species such as winter annual plants (Kimball et al., 2010). Seasonal changes warp the temporal template upon which life histories unfold because life‐histories are extremely time‐dependent and balanced by trade‐offs between current and future allocations crucial for fitness (e.g. timing of reproduction). Evolutionary consequences of seasonality expansions are not systematically understood: for example, phenologies of species (the timing of life‐history events such as flowering) are shifting incongruently and unpredictably. Dramatic shifts and variability of phenological responses documented worldwide suggest that the underlying cyclicity of the environment plays an important role in shaping life‐history trait distributions in ways that we do not understand well yet. Comparing species with different generation times experiencing the same directional change in seasonality should be enlightening for understanding divergent responses in life‐history distributions as a function of the ratio between generation time and seasonality change.

Of crucial importance for understanding life‐history evolution in cyclical environments, particularly in populations with overlapping generations, is that disturbance events are not necessarily ‘selection events’ in the traditional sense because mortality incurred by events are non‐selective with respect to life‐history strategy. For instance, juveniles of the same age or stage killed by a disturbance may have taken very different amounts of time to mature to that point (via low vs. high μ). Selectively advantageous vital rates, instead, are determined by integrating the realised costs and benefits of life‐history allocations in the context of recent and forthcoming fluctuations over entire lifetimes and over generations. If those fluctuations are predictable, then over multiple generations the ultimate ‘winning’ strategy and distribution around it emerges. When environmental fluctuations are on much longer timescales than generations, fluctuations can impose ‘fluctuating selection’, where the population adaptively tracks the environment over generations (Bell, 2010; Hairston et al., 2005), or induce transgenerational plasticity, where environments experienced by a generation modify expressed phenotypes of subsequent generations (Galloway & Etterson, 2007; Walsh et al., 2016). When generation times are much longer than periodicities of environmental fluctuation, physiological or behavioural plasticity that mediate fluctuations on much shorter timescales may be targets of selection (Gross et al., 2010; Lande, 2014; Moran, 1992; Scheiner et al., 2020). However, our investigations are in the realm in which generation time is comparable to environmental fluctuations. Relative time scale similarity of life history and environmental fluctuations is characteristic of many birds, insects and annual plant systems. In such cases, we argue, the environmental fluctuation regimes—defined by parameters like periodicity—are agents of selection themselves that shape life‐history distributions in populations.

## AUTHORS CONTRIBUTIONS

JSP conceived the theoretical ideas, designed and coded agent‐based models, designed and executed the experiment, analysed data and wrote the manuscript; JTW contributed to model and experiment design, analysis and manuscript writing.

### PEER REVIEW

The peer review history for this article is available at https://publons.com/publon/10.1111/ele.13867.

### Open Research Badges

This article has earned an Open Data Badge for making publicly available the digitally‐shareable data necessary to reproduce the reported results. The data is available at Dryad: https://doi.org/10.5061/dryad.kkwh70s58 and GitHub: https://github.com/john‐s‐park/SlowerCycles_LHVar.

## Supporting information

Supplementary Material

## Data Availability

All raw data files and R code are archived in Dryad (DOI: https://doi.org/10.5061/dryad.kkwh70s58) and also openly available at https://github.com/john‐s‐park/SlowerCycles_LHVar.
