# Supplementary material for: Slower environmental cycles maintain greater life‐history variation within populations
Source: Ecol Lett. 2021 Sep 2;24(11):2452–63. doi: 10.1111/ele.13867 (PMC9292183; doi:10.1111/ele.13867)
Supplement: Supplementary file 1 — Supplementary Material [file ELE-24-2452-s001.docx]

SUPPORTING INFORMATION for:

**Slower environmental cycles increase life-history variation within populations**

John S. Park^1^ & J. Timothy Wootton^2^

^1^Committee on Evolutionary Biology, University of Chicago

Zoology 400, 1101 E. 57^th^ Street, Chicago, IL 60637

^2^Department of Ecology & Evolution, University of Chicago

Corresponding author:

John S. Park

johnspark@uchicago.edu

**TABLE OF CONTENTS**

| **S1** Derivation: deterministic model of life history fitness across cycle periods | 1-2 |
| --- | --- |
| **S2** Simulation schematic | 3 |
| **S3** Experimental schematic | 4 |
| **S4** Permutation distributions of experimental intrapopulation variance differences between treatments | 5-6 |
| **S5** Phenotypic means in experimental populations | 7 |
| **S6** Deterministic *vs.* stochastic simulation variance trajectories | 8-9 |
| **S7** Population size and structure dynamics in simulations | 10 |

**S1. Derivation: deterministic model of life history fitness across cycle periods**

The following is an abridged derivation of deterministic asymptotic growth ($\lambda$) as a function of life history traits and environment cycle periodicity developed in Park (2019). This calculation was used to construct fitness landscapes and profiles in Fig. 1 of the main text of this paper.

First, consider the continuous dynamics of juveniles (J) and adults (A) of a population in a constant environment:

|  | $\frac{dJ}{dt} = -(\mu+d)J+fA$  $\frac{dA}{dt} = \mu J-\gamma A$ |
| --- | --- |

where $\mu$ is juvenile maturation rate, *d* is background juvenile mortality rate, *f* is adult fecundity, and $\gamma$ is background adult mortality rate. This system of ODE’s, in matrix form, can be expressed as:

|  | $\mathbf{M}=\left[ \begin{matrix} -(\mu+d) & f \\ \mu& -\gamma\end{matrix} \right]$ |
| --- | --- |

The solution of stage-structure dynamics of this population can be given as a function of the eigenvalue and eigenvector elements of matrix **M** where $v_{(i)j}$ is the $j^{th}$ element of eigenvector *i*, associated with eigenvalue $\lambda_{i}$ of $\mathbf{M}$:

$$J\left( t \right)= \frac{A\left( 0 \right)v_{\left( 2 \right)1}-J(0)v_{\left( 2 \right)2}}{v_{\left( 2 \right)1}v_{\left( 1 \right)2}- v_{\left( 2 \right)2}v_{\left( 1 \right)1}}v_{\left( 1 \right)1}e^{\lambda_{1}t}+ \frac{J\left( 0 \right)v_{\left( 1 \right)2}-A(0)v_{\left( 1 \right)1}}{v_{\left( 2 \right)1}v_{\left( 1 \right)2}- v_{\left( 2 \right)2}v_{\left( 1 \right)1}}v_{\left( 2 \right)1}e^{\lambda_{2}t}$$

$$A\left( t \right)= \frac{A\left( 0 \right)v_{\left( 2 \right)1}-J(0)v_{\left( 2 \right)2}}{v_{\left( 2 \right)1}v_{\left( 1 \right)2}- v_{\left( 2 \right)2}v_{\left( 1 \right)1}}v_{\left( 1 \right)2}e^{\lambda_{1}t}+ \frac{J\left( 0 \right)v_{\left( 1 \right)2}-A(0)v_{\left( 1 \right)1}}{v_{\left( 2 \right)1}v_{\left( 1 \right)2}- v_{\left( 2 \right)2}v_{\left( 1 \right)1}}v_{\left( 2 \right)2}e^{\lambda_{2}t}$$

Next, let $J_{w}(t)$ and $A_{w}(t)$ describe solutions of the two stages before the first disturbance, and $J_{w+1}(t)$ and $A_{w+1}(t)$ before the second disturbance, and so on. Let *T* = deterministic length of the interval between periodic disturbance events. Therefore, $J_{w}(T)$ and $A_{w}(T)$ give abundances of the stages immediately before the first disturbance, and $J_{w+1}(0)$ and $A_{w+1}(0)$ immediately after the first disturbance, and so on. Let $S_{J}$ and $S_{A}$ describe *survival rates* of each stage whenever disturbance occurs, such that:

|  | $J_{w+1}\left( 0 \right)= S_{J}J_{w}\left( T \right)$  $A_{w+1}\left( 0 \right)= S_{A}A_{w}(T)$ |  |
| --- | --- | --- |

Let $N$be the vector of abundances of the two stages immediately after disturbance, and **P** the matrix that projects $N_{w}$to $N_{w+1}$.

$$\begin{matrix} {\overset{⃗}{N}}_{w+1} & =\left[ \begin{matrix} S_{J}J_{w}(T) \\ S_{A}A_{w}(T) \end{matrix} \right]=\mathbf{P}{\overset{⃗}{N}}_{w} \\ & =\left[ \begin{matrix} S_{J}[\frac{A_{w}(0)v_{(2)1}-J_{w}(0)v_{(2)2}}{v_{(2)1}v_{(1)2}-v_{(2)2}v_{(1)1}}v_{(1)1}e^{\lambda_{1}T}+\frac{J_{w}(0)v_{(1)2}-A_{w}(0)v_{(1)1}}{v_{(2)1}v_{(1)2}-v_{(2)2}v_{(1)1}}v_{(1)2}e^{\lambda_{2}T}] \\ S_{A}[\frac{A_{w}(0)v_{(2)1}-J_{w}(0)v_{(2)2}}{v_{(2)1}v_{(1)2}-v_{(2)2}v_{(1)1}}v_{(1)2}e^{\lambda_{1}T}+\frac{J_{w}(0)v_{(1)2}-A_{w}(0)v_{(1)1}}{v_{(2)1}v_{(1)2}-v_{(2)2}v_{(1)1}}v_{(2)2}e^{\lambda_{2}T}] \end{matrix} \right]=\mathbf{P}\left[ \begin{matrix} J_{w}(0) \\ A_{w}(0) \end{matrix} \right] \\ & =\left[ \begin{matrix} \frac{J_{w}(0)S_{J}[(v_{(1)2}e^{\lambda_{2}T}v_{(1)2}-v_{(1)1}e^{\lambda_{1}T}v_{(2)2})]}{v_{(2)1}v_{(1)2}-v_{(2)2}v_{(1)1}}+\frac{A_{w}(0)S_{J}[(v_{(1)1}e^{\lambda_{1}T}v_{(2)1}-v_{(1)2}e^{\lambda_{2}T}v_{(1)1})]}{v_{(2)1}v_{(1)2}-v_{(2)2}v_{(1)1}} \\ \frac{J_{w}(0)S_{A}[(v_{(2)2}e^{\lambda_{2}T}v_{(1)2}-v_{(1)2}e^{\lambda_{1}T}v_{(2)2})]}{v_{(2)1}v_{(1)2}-v_{(2)2}v_{(1)1}}+\frac{A_{w}(0)S_{A}[(v_{(1)2}e^{\lambda_{1}T}v_{(2)1}-v_{(2)2}e^{\lambda_{2}T}v_{(1)1})]}{v_{(2)1}v_{(1)2}-v_{(2)2}v_{(1)1}} \end{matrix} \right]=\mathbf{P}\left[ \begin{matrix} J_{w}(0) \\ A_{w}(0) \end{matrix} \right] \end{matrix}$$

Therefore,

|  | $\mathbf{P}\boldsymbol{=}\left[ \begin{matrix} \boldsymbol{S}_{\boldsymbol{J}}\frac{\boldsymbol{[(}\boldsymbol{v}_{\boldsymbol{(1)2}}\boldsymbol{e}^{\boldsymbol{\lambda}_{\boldsymbol{2}}\boldsymbol{T}}\boldsymbol{v}_{\boldsymbol{(1)2}}\boldsymbol{-}\boldsymbol{v}_{\boldsymbol{(1)1}}\boldsymbol{e}^{\boldsymbol{\lambda}_{\boldsymbol{1}}\boldsymbol{T}}\boldsymbol{v}_{\boldsymbol{(2)2}}\boldsymbol{)]}}{\boldsymbol{v}_{\boldsymbol{(2)1}}\boldsymbol{v}_{\boldsymbol{(1)2}}\boldsymbol{-}\boldsymbol{v}_{\boldsymbol{(2)2}}\boldsymbol{v}_{\boldsymbol{(1)1}}} & \boldsymbol{S}_{\boldsymbol{J}}\frac{\boldsymbol{[(}\boldsymbol{v}_{\boldsymbol{(1)1}}\boldsymbol{e}^{\boldsymbol{\lambda}_{\boldsymbol{1}}\boldsymbol{T}}\boldsymbol{v}_{\boldsymbol{(2)1}}\boldsymbol{-}\boldsymbol{v}_{\boldsymbol{(1)2}}\boldsymbol{e}^{\boldsymbol{\lambda}_{\boldsymbol{2}}\boldsymbol{T}}\boldsymbol{v}_{\boldsymbol{(1)1}}\boldsymbol{)]}}{\boldsymbol{v}_{\boldsymbol{(2)1}}\boldsymbol{v}_{\boldsymbol{(1)2}}\boldsymbol{-}\boldsymbol{v}_{\boldsymbol{(2)2}}\boldsymbol{v}_{\boldsymbol{(1)1}}} \\ \boldsymbol{S}_{\boldsymbol{A}}\frac{\boldsymbol{[(}\boldsymbol{v}_{\boldsymbol{(2)2}}\boldsymbol{e}^{\boldsymbol{\lambda}_{\boldsymbol{2}}\boldsymbol{T}}\boldsymbol{v}_{\boldsymbol{(1)2}}\boldsymbol{-}\boldsymbol{v}_{\boldsymbol{(1)2}}\boldsymbol{e}^{\boldsymbol{\lambda}_{\boldsymbol{1}}\boldsymbol{T}}\boldsymbol{v}_{\boldsymbol{(2)2}}\boldsymbol{)]}}{\boldsymbol{v}_{\boldsymbol{(2)1}}\boldsymbol{v}_{\boldsymbol{(1)2}}\boldsymbol{-}\boldsymbol{v}_{\boldsymbol{(2)2}}\boldsymbol{v}_{\boldsymbol{(1)1}}} & \boldsymbol{S}_{\boldsymbol{A}}\frac{\boldsymbol{[(}\boldsymbol{v}_{\boldsymbol{(1)2}}\boldsymbol{e}^{\boldsymbol{\lambda}_{\boldsymbol{1}}\boldsymbol{T}}\boldsymbol{v}_{\boldsymbol{(2)1}}\boldsymbol{-}\boldsymbol{v}_{\boldsymbol{(2)2}}\boldsymbol{e}^{\boldsymbol{\lambda}_{\boldsymbol{2}}\boldsymbol{T}}\boldsymbol{v}_{\boldsymbol{(1)1}}\boldsymbol{)]}}{\boldsymbol{v}_{\boldsymbol{(2)1}}\boldsymbol{v}_{\boldsymbol{(1)2}}\boldsymbol{-}\boldsymbol{v}_{\boldsymbol{(2)2}}\boldsymbol{v}_{\boldsymbol{(1)1}}} \end{matrix} \right]$ |
| --- | --- |

The dominant eigenvalue of **P** (referred to as $\lambda$for simplicity) gives the asymptotic growth rate of a genotype with a life history strategy defined by the traits in **M**, living in an environment defined by deterministic periodicity T. The fitness profile of life history strategies in a particular environment is computed by calculating $\lambda$’s across combinations of life history traits, holding T constant. Note that combinations are constrained by biologically realistic trade-offs among the traits. Comparative fitness profiles between environments, for example between Fast and Slow regimes as discussed in the main text, are computed by manipulating the value of T. A full fitness landscape is computed by sweeping through a continuous gradient of T.

**S2.** **Simulation schematic**


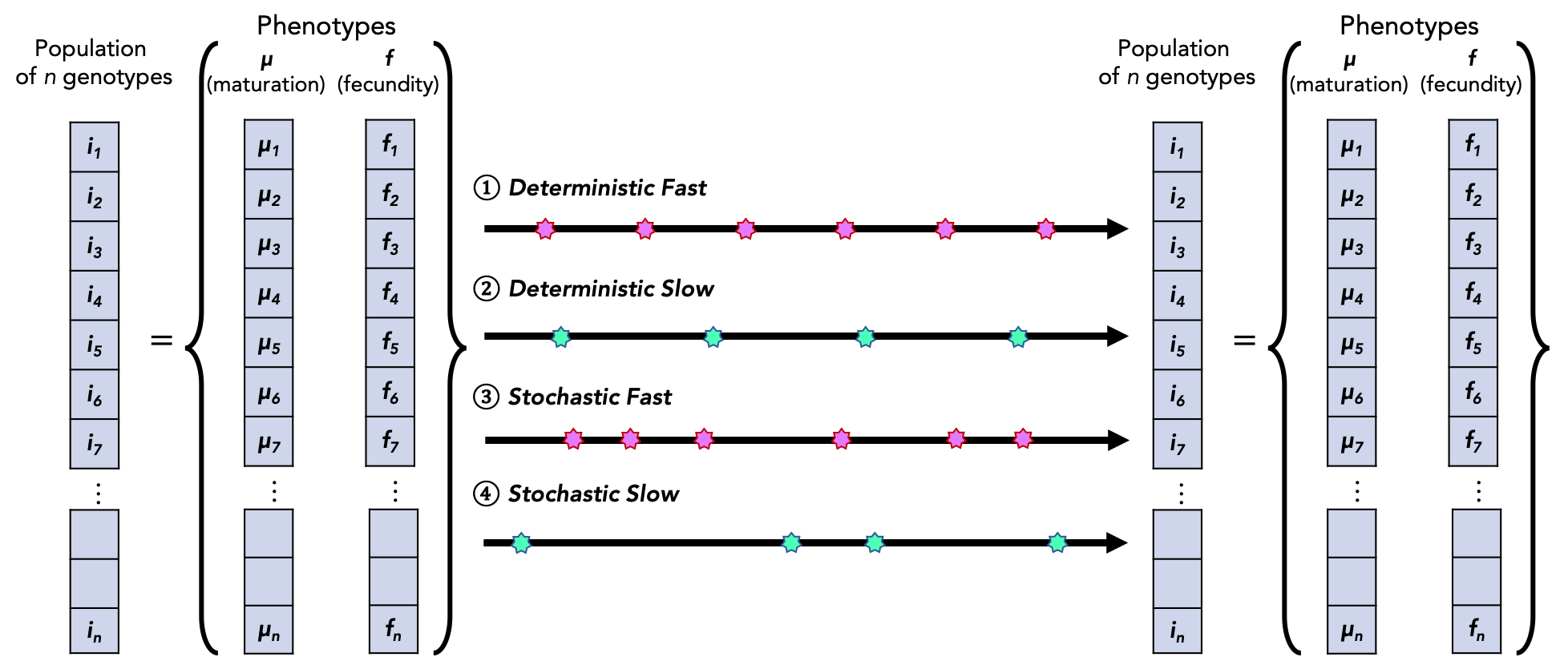


Simulated populations are vectors of individuals, whose identities are defined by a pair of phenotypes $\mu$ (maturation rate) and $f$ (fecundity). Each individual’s continuous state [0,3.0] is tracked throughout the simulation. Individuals grow, reproduce, and die following the below rules, and the population vector grows and shrinks through time accordingly.

**Demographic dynamics**

1. Individuals grow at their innate maturation rates ($\mu$).
2. Individuals become mature at a certain point (state = 1.0), and start reproducing (adding new individuals to the population vector.
3. Offspring phenotypes are copies of the parent’s (more specifically, their mother’s, since mating is not modelled) with some error (e.g. recombination, mutation).
4. Juvenile maturation rate ($\mu$) has a linear trade-off with juvenile intrinsic survival per time step (i.e. higher $\mu$ = higher mortality probability), and adult fecundity ($f$) has a linear trade-off with adult intrinsic survival (i.e. higher $f$ = higher mortality probability).
5. Nonlinear density-dependent mortality applies equally across all individuals of all states.
6. Individuals senesce and die (excised from population vector) when they reach state = 3.0.

**Periodic disturbance-induced death**

1. The external environment disturbs the population, introducing stage-structure perturbation (i.e. heightened juvenile mortality) mirroring what happens in nature as well as what was administered in the lab experiment.
2. Four regimes were investigated as shown above – deterministic Fast, deterministic Slow, and stochastic analogs of each wherein the number of disturbances within the duration of the simulation was equal to their counterparts but intervals were random.

To study the dynamics of means and variances of phenotypes within simulated populations, we tracked the distributions of phenotypes represented by individuals of all states through time.

**S3.** **Experimental schematic**


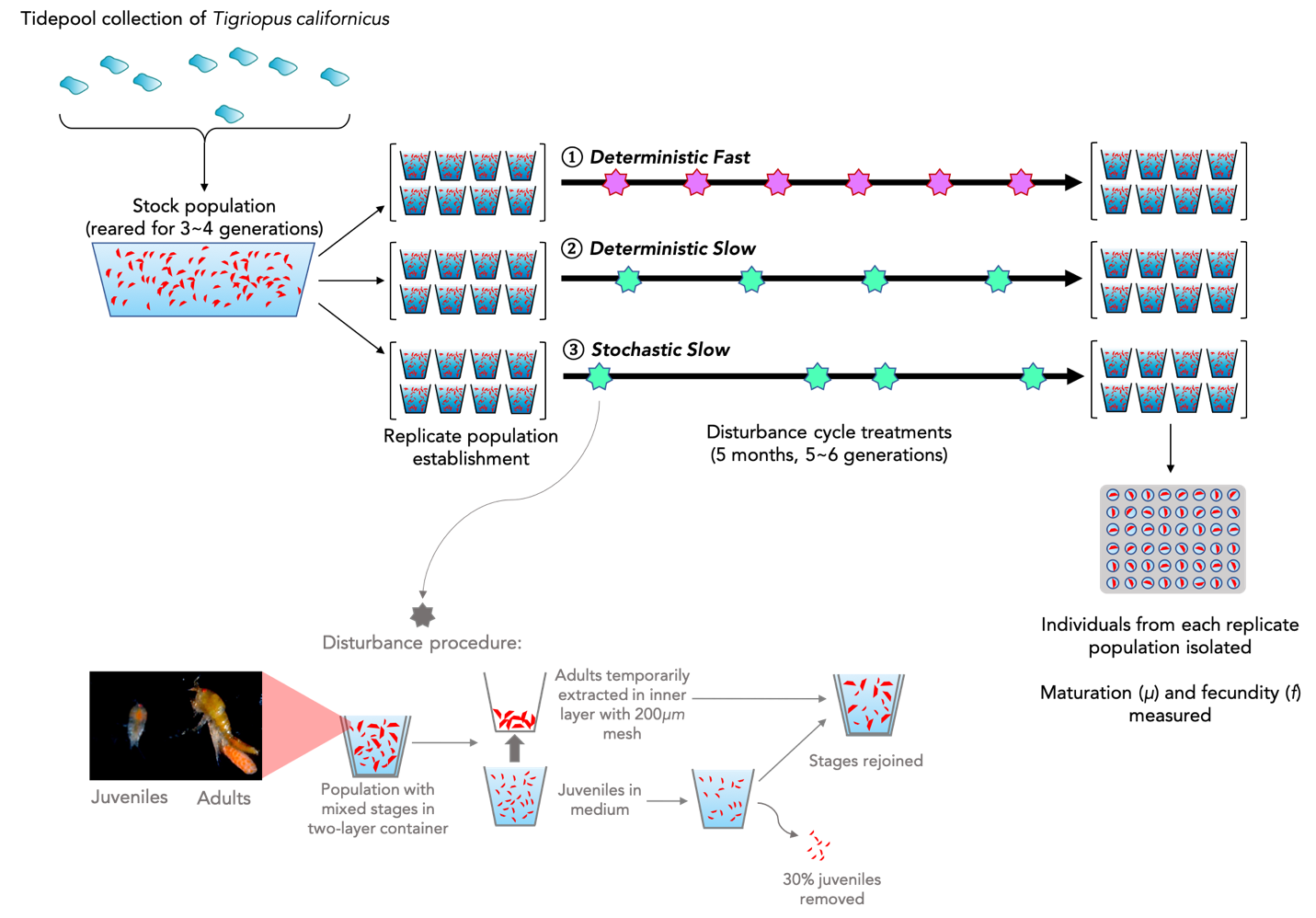


**S4. Permutation distributions of experimental population variances**


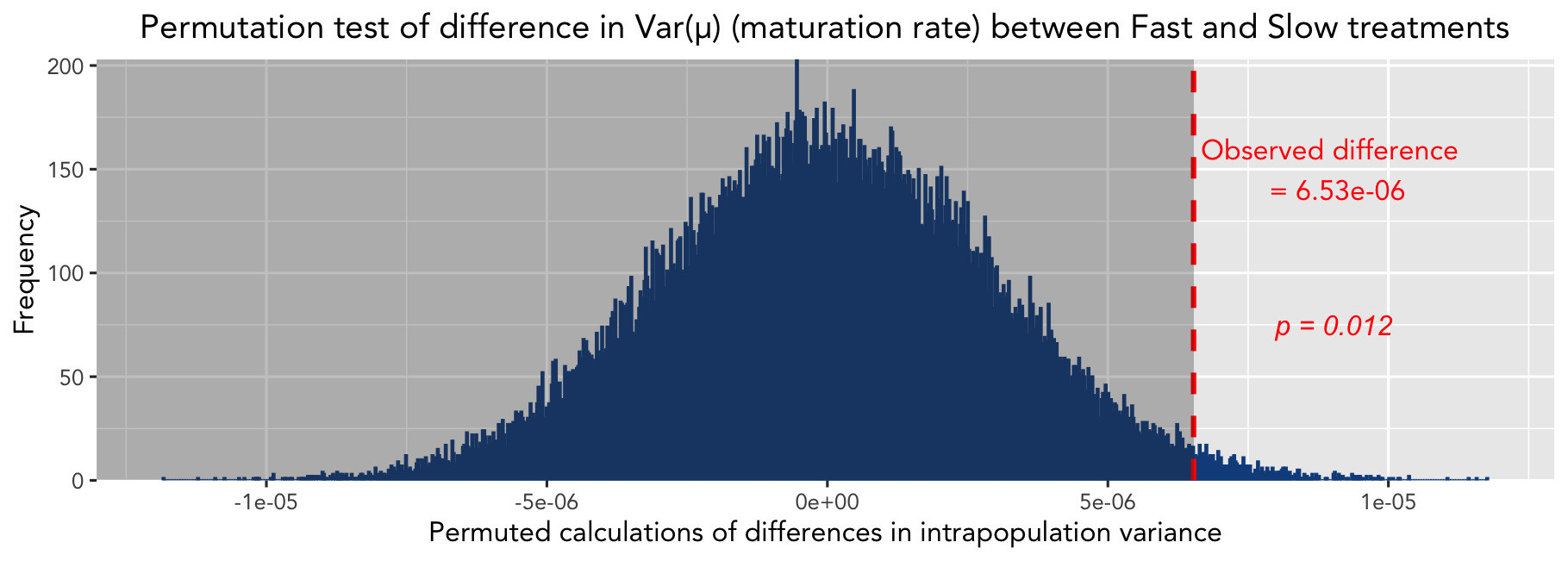


**Fig. S4.1** Permutation distribution used to calculate the p-value of the disparity between intrapopulation maturation rate variances of deterministic Fast and Slow treatment populations.


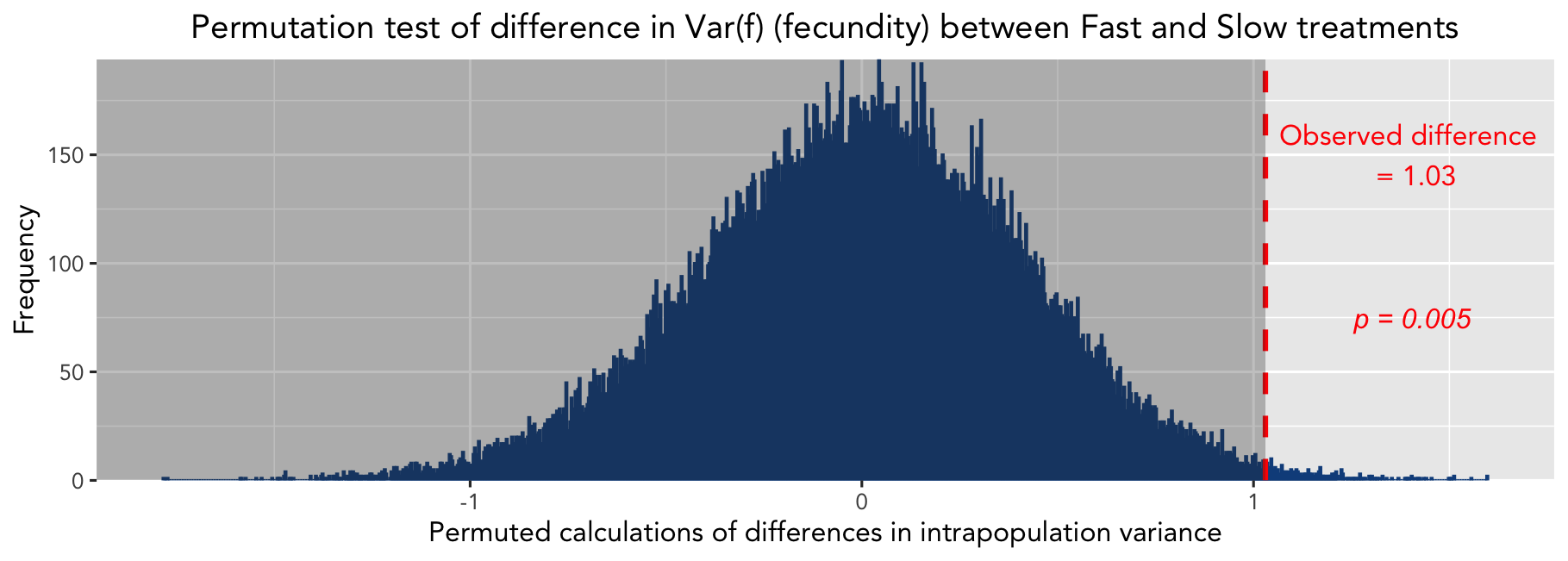


**Fig. S4.2** Permutation distribution used to calculate the p-value of the disparity between intrapopulation fecundity variances of deterministic Fast and Slow treatment populations.


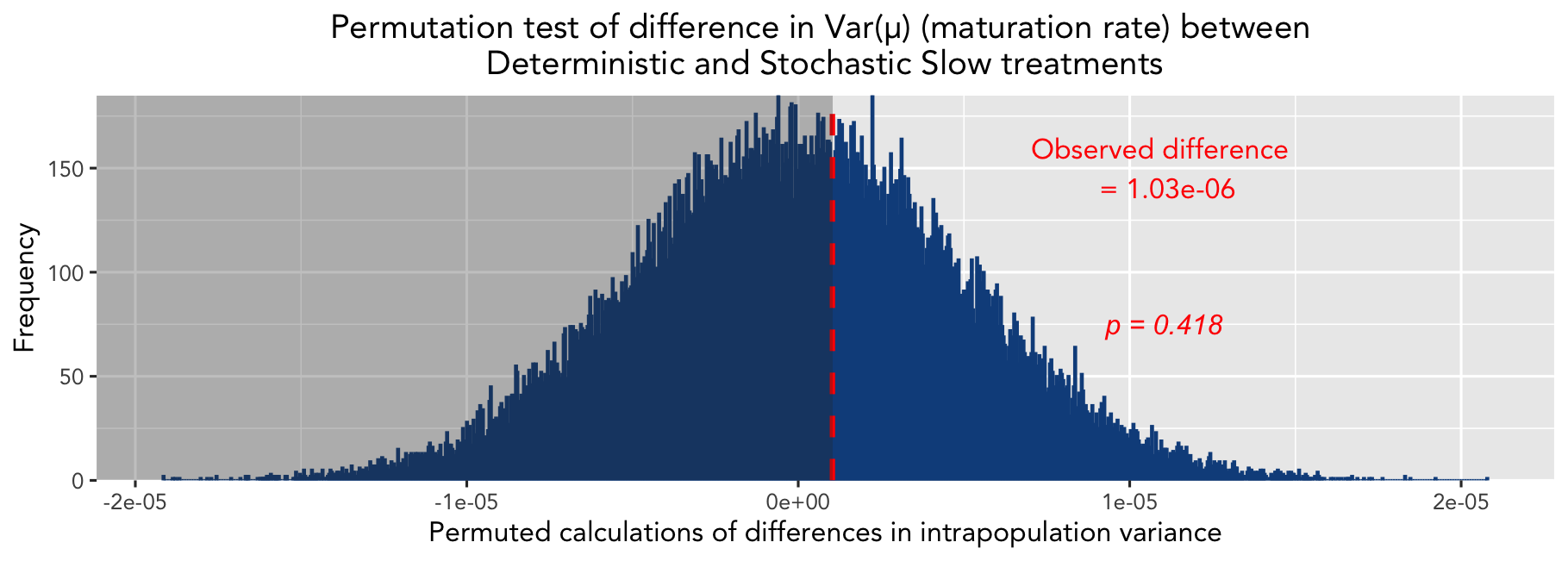


**Fig. S4.3** Permutation distribution used to calculate the p-value of the disparity between intrapopulation maturation rate variances of deterministic and stochastic Slow treatment populations.


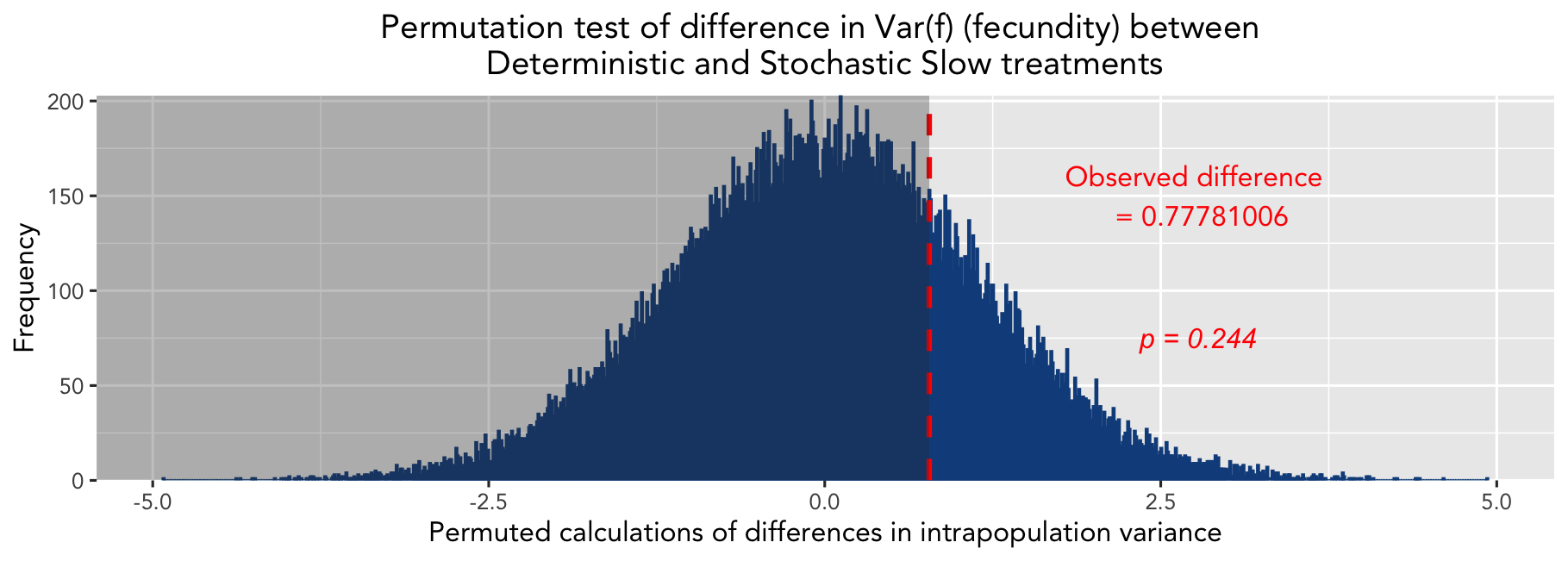


**Fig. S4.4** Permutation distribution used to calculate the p-value of the disparity between intrapopulation fecundity variances of deterministic and stochastic Slow treatment populations.

**S5.** **Phenotypic means in experimental populations**


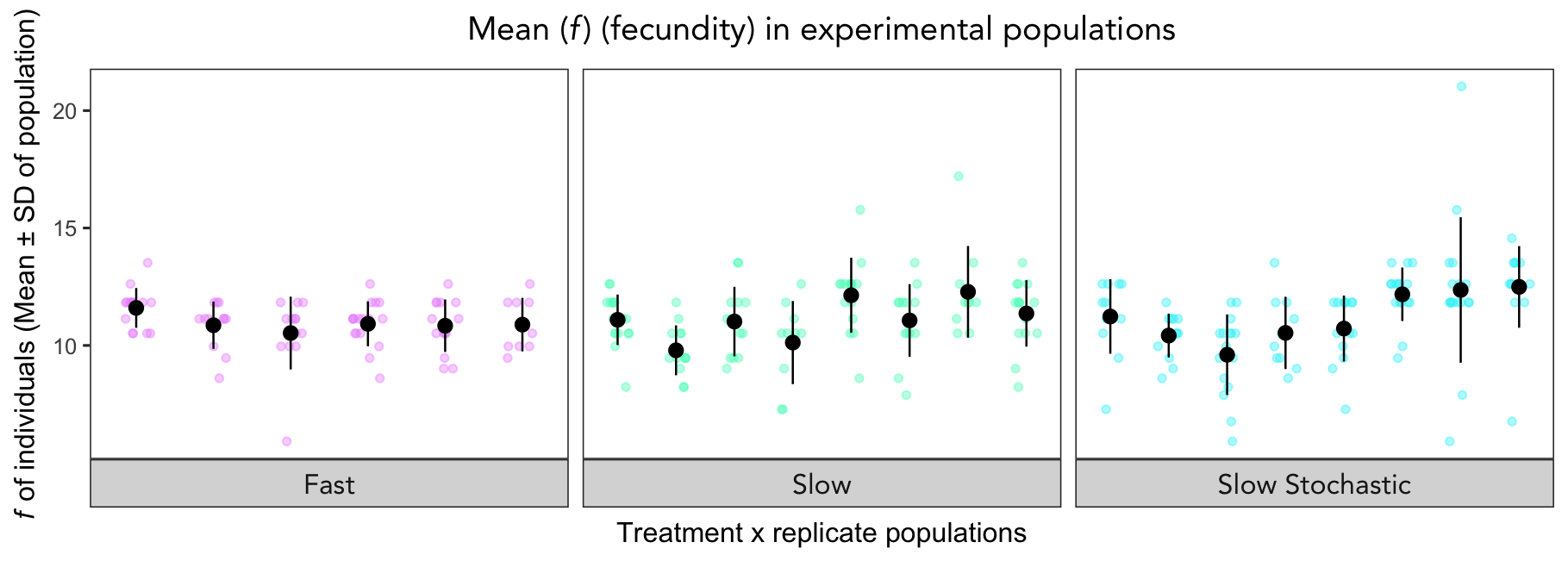


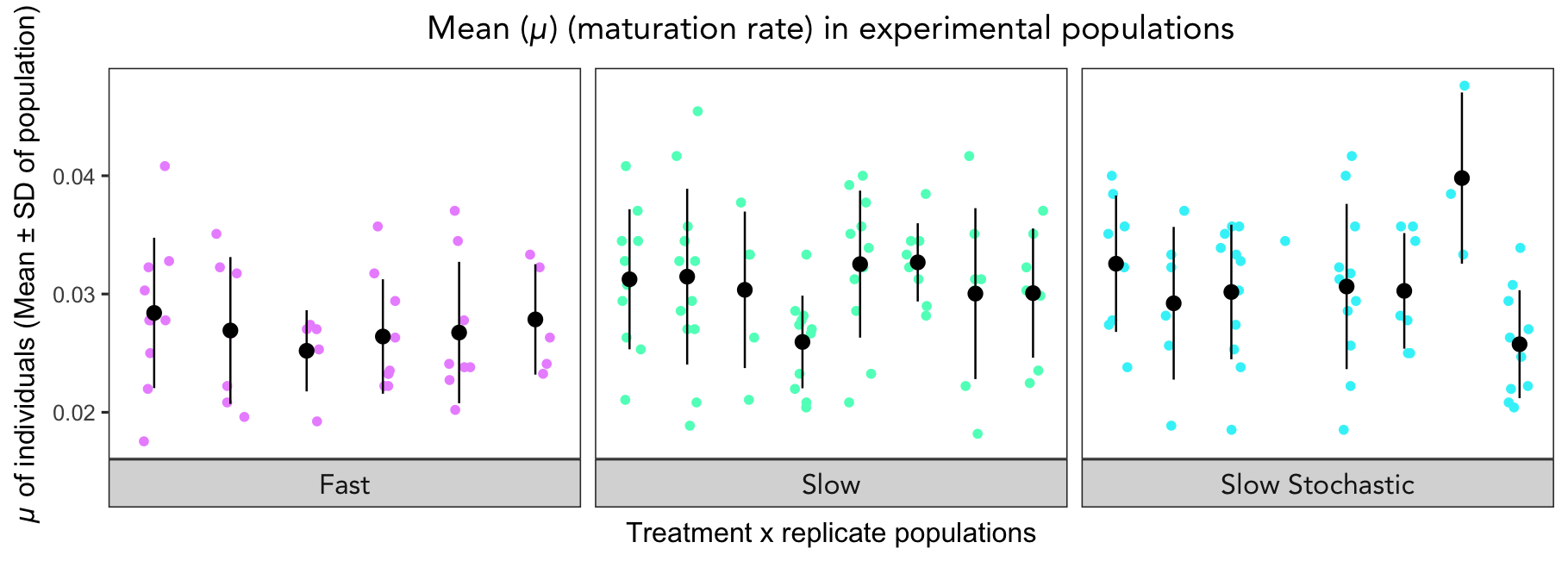


**Fig. S5.1** Phenotypic measurements from selection experiment. Colored scatter points show individual measurements, and black dots with bars show mean ± sd of each replicate population. Panels show replicate populations of each treatment.

| Trait | Treatment comparison | p-value |
| --- | --- | --- |
| **f**  (reproductive rate) | Fast vs. Slow | 0.345 |
|  | Deterministic Slow vs. Stochastic Slow | 0.879 |
| $\boldsymbol{\mu}$  (maturation rate) | **Fast vs. Slow** | **0.013** |
|  | Deterministic Slow vs. Stochastic Slow | 0.879 |

**Table. S5.1** Tests of differences in phenotypic means at the replicate population level. P-values are from two-tailed nonparametric Mann-Whitney tests using population means (black dots in Fig. S4.1).

**S6. Deterministic *vs.* stochastic variance trajectories and endpoints**

**
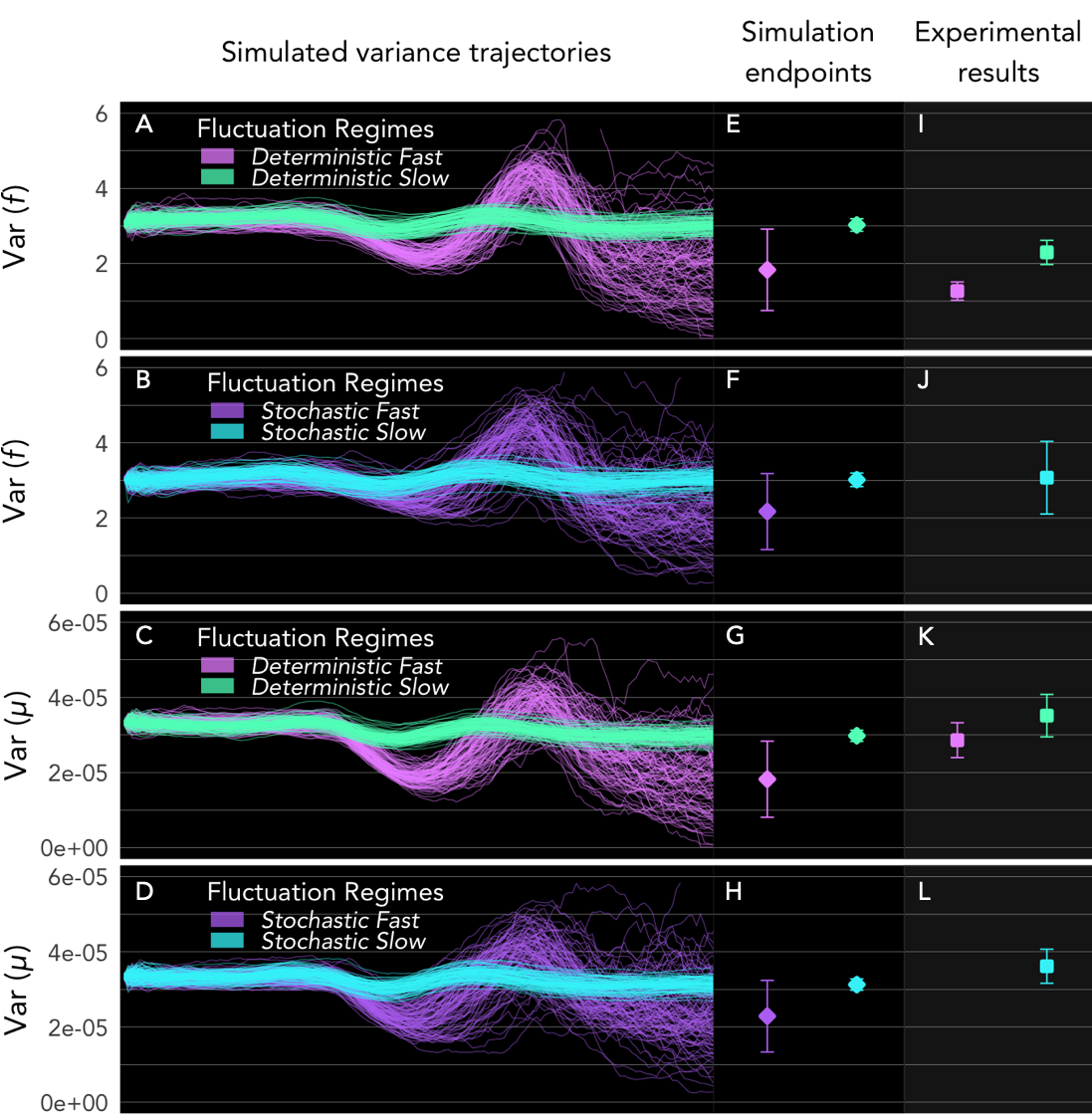
**

**Fig. S6.1** Intrapopulation variance trajectories of fecundity (*f)* and maturation rate ($\mu$) in deterministic and stochastic simulation iterations. The deterministic panels (first and third rows; panels A, E & I, and C, G & K) are identical to Fig. 4 of main text. The second and fourth rows (panels B, F & J, and D, H & L) are the stochastic analogs. Note that in panels J and L, there is only one treatment, Stochastic Slow.


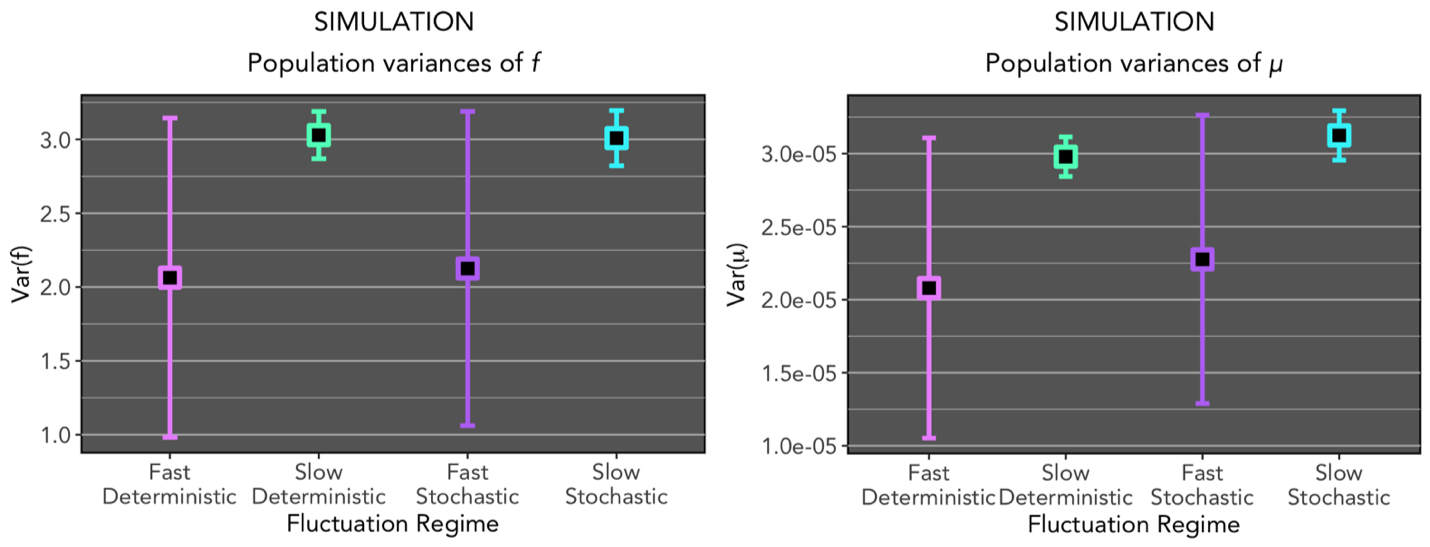


**Fig. S6.2** Simulation endpoint variances (identical data as second column of Fig. S6.1, presented for easier visual comparisons across treatments).

| Trait | Simulation regime variance comparison | T-statistic | p-value |
| --- | --- | --- | --- |
| **f**  (fecundity) | **Deterministic Fast vs. Deterministic Slow** | **6.307** | **<0.001** |
|  | **Stochastic Slow vs. Stochastic Fast** | **8.397** | **<0.001** |
|  | Deterministic Slow vs. Stochastic Slow | 0.904 | 0.368 |
|  | Deterministic Fast vs. Stochastic Fast | -1.209 | 0.229 |
| $\boldsymbol{\mu}$  (maturation rate) | **Deterministic Fast vs. Deterministic Slow** | **6.289** | **<0.001** |
|  | **Stochastic Slow vs. Stochastic Fast** | **8.549** | **<0.001** |
|  | **Deterministic Slow vs. Stochastic Slow** | **-6.72** | **<0.001** |
|  | **Deterministic Fast vs. Stochastic Fast** | **-2.071** | **0.04** |

**Table S6.1.** Comparisons of intrapopulation phenotypic variances between simulation realizations. Welch’s two-sample t-tests with log-transformed measures of variance showed strong differences between Fast and Slow regimes for both traits, both in statistical significance and magnitude. Deterministic and stochastic analogs produced smaller, and less significant differences in intrapopulation variance overall (though more significant for $\mu$ than *f*).

**S7.** **Population structure and size dynamics in simulations**

**Fig. S7.1** Stage-structure dynamics in deterministically and stochastically slow and fast regimes in one representative realization of the simulation.

**Fig. S7.2** Population size dynamics in deterministically and stochastically slow and fast regimes in one representative realization of the simulation.
